# Spontaneous tumor lysis syndrome in hyperaggressive mantle cell lymphoma

**DOI:** 10.1002/jha2.531

**Published:** 2022-08-04

**Authors:** James Wilson

**Affiliations:** ^1^ Department of Haematology Pinderfields General Hospital Wakefield UK

1

An otherwise well 61‐year‐old male presented to his primary care practitioner with drenching sweats, weight loss, massive splenomegaly (30 cm as measured by computed tomography [CT], Figure 1A) and a lymphocytosis (white blood cell [WBC] at diagnosis 150 × 10^9^/L). The peripheral smear was notable for the presence of pleomorphic abnormal lymphocytes, with clumped chromatin, high nuclear:cytoplasm ratio and irregular nuclei (Figure 1B). Peripheral blood flow cytometry revealed a kappa light chain restricted population of mature B‐cells which co‐expressed CD20 and CD5 (Figure 1C). CD23 and CD10 were negative. Fluorescent in situ hybridization revealed a *CCND1/IGH* rearrangement, consistent with a diagnosis of advanced stage mantle cell lymphoma (MCL). Approximately 3 weeks after diagnosis, he was admitted electively for chemotherapy.

**FIGURE 1:**
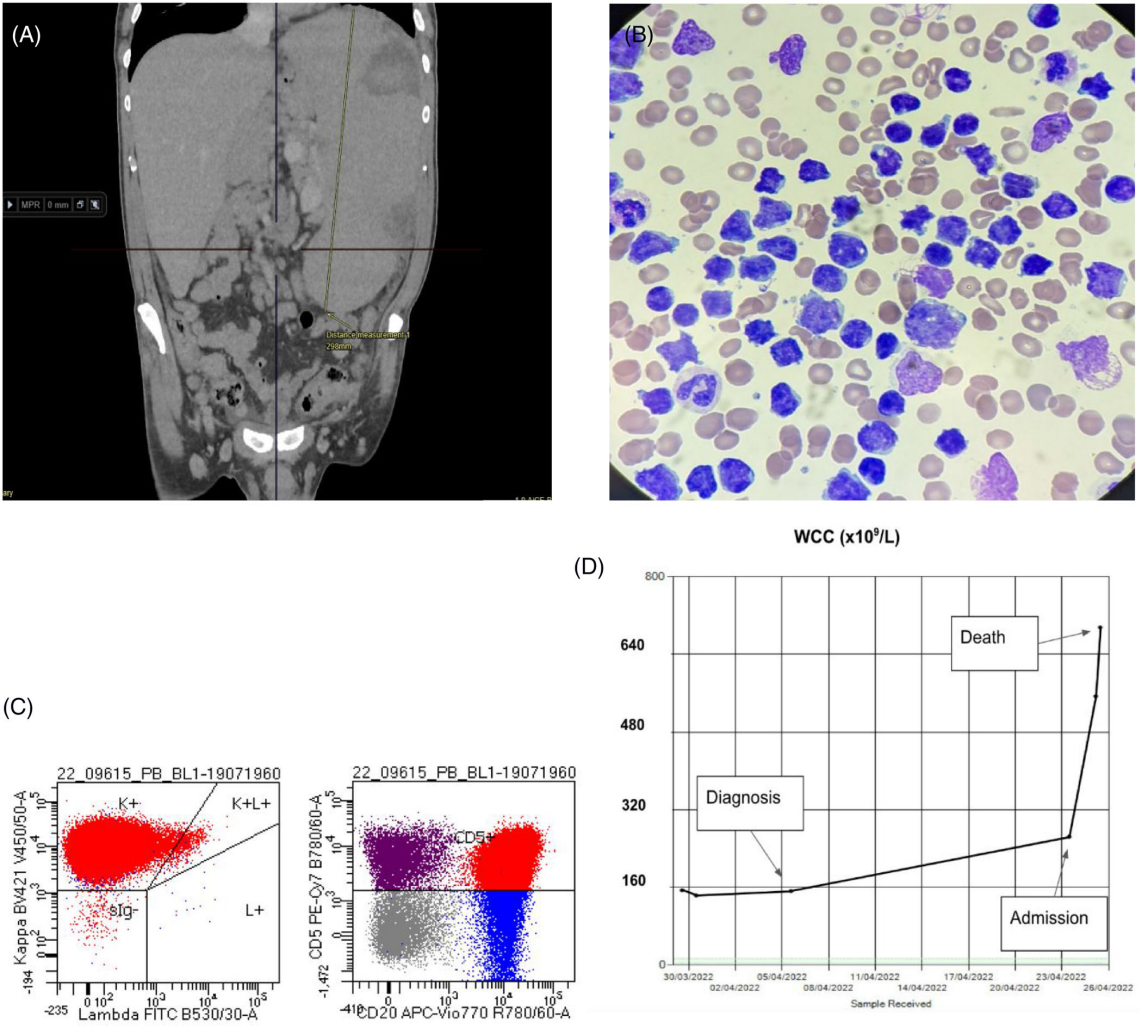
(A) CT demonstrated massive splenomegaly measuring 30 cm at maximum diameter. (B) Pleomorphic abnormal lymphocytes with condensed chromatin and irregular nuclei. (C) Flow cytometry demonstrated a kappa light chain restricted population of CD20 and CD5 positive lymphocytes. (D) Explosive increase in WBC count coincided with the onset of spontaneous tumour lysis syndrome.

On admission to the unit he was well, with normal vital signs and a normal baseline tumor lysis profile (K, Phos, Ca, urate and creatinine all within institution limits). His WBC was noted to have increased modestly to 264 × 109/L. Unfortunately, over the course of the next 12 h, prior to the administration of any systemic anti–cancer therapy (including steroids), he developed spontaneous tumor lysis syndrome and died of catastrophic metabolic derangement, despite aggressive supportive care. He developed severe, refractory hyperkalemia (7.5 mmol/L), severe hyperphosphatemia (3.83 mmol/L) and profound metabolic acidosis (pH <6.9, HCO_3_^–^ 3 mmol/L, lactate 17 mmol/L). His WBC had also climbed explosively to 695 × 10^9^/L (Figure 1D), putting him at significant risk of leukostasis.

I postulate that, via clonal evolution, his disease acquired a hyperproliferative phenotype, resulting in fulminant, spontaneous tumor lysis syndrome. There are limited reports of secondary translocations involving the *MYC* proto‐oncogene in patients with MCL. This is associated with aggressive clinical course and short overall survival; however there are no reports of MCL behaving this aggressively in the literature. Practitioners should be aware of this rare but potentially devastating complication of MCL.

## AUTHOR CONTRIBUTION

James Wilson wrote the manuscript and took pictures.

## CONFLICT OF INTEREST

The author declares they have no conflicts of interest.

## FUNDING INFORMATION

The author received no specific funding for this work.

## ETHICS STATEMENT

No research on human was performed on this study.

## Data Availability

Data sharing not applicable—no new data generated.

